# Laser Lead Extraction During Venoarterial ECMO support

**DOI:** 10.21470/1678-9741-2020-0391

**Published:** 2022

**Authors:** Yalin Yildirim, Johannes Petersen, Tobias Tönnis, Christian Detter, Hermann Reichenspurner, Simon Pecha

**Affiliations:** 1 Department of Cardiovascular Surgery, University Heart and Vascular Center Hamburg, Hamburg, Germany.; 2 Department of Cardiology/Electrophysiology, University Heart and Vascular Center Hamburg, Hamburg, Germany.

**Keywords:** Extracorporeal Membrane Oxygenation, Stroke Volume, Cardiopulmonary Bypass, Ventricular Function, Left, Heart Arrest, Endocarditis, Sepsis

## Abstract

The treatment of valvular endocarditis in patients with cardiac implantable electrophysiological device (CIED) includes valvular surgery and lead extraction. This can be challenging in patients with severely reduced left ventricular ejection fraction (LVEF). Reduced LVEF in combination with sepsis and cardioplegic cardiac arrest can make weaning from cardiopulmonary bypass difficult. Some of these patients require venoarterial extracorporeal membrane oxygenation (VA-ECMO) for postcardiotomy syndrome. Lead extraction by manual traction is often not possible in cases with a long lead dwell time. Therefore, a lead extraction procedure with powered sheaths is required during the VA-ECMO support. We describe our technique for laser lead extraction during VA-ECMO support in a 64-year-old patient with triple valve endocarditis and lead vegetations.

**Table:** Abbreviations, acronyms & symbols

AV	= Atrioventricular
CIED	= Cardiac implantable electrophysiological device
CRT	= Cardiac resynchronization therapy
ECMO	= Extracorporeal membrane oxygenation
ICD	= Implantable cardioverter-defibrillator
ICU	= Intensive care unit
LLD	= Lead locking device
LVEF	= Left ventricular ejection fraction
OR	= Operating room
RV	= Right ventricle
SVC	= Superior vena cava
VA-ECMO	= Venoarterial extracorporeal membrane oxygenation

## INTRODUCTION

In recent years, the number of pacemaker and implantable cardioverter-defibrillator (ICD) implantations has been rising. In line with this, an increasing number of patients require lead revision and extraction due to several reasons^[1]^. In guidelines, there are several class I recommendations for lead extraction, including local and systemic infections, as well as lead endocarditis. In patients with lead endocarditis, complete removal of all lead material from the vascular space and antibiotic treatment is recommended^[2-4]^. In cases with valvular involvement, additional surgical valve repair or replacement is often necessary^[5]^. Especially in cases of prosthetic valve endocarditis, valve surgery is needed. In cases of short implant duration of the cardiac implantable electrophysiological device (CIED), the leads can be removed by manual traction during valve surgery. However, in cases with long implant duration, lead extraction with specialized tools is necessary. Here, rotational mechanical sheaths as well as laser sheaths can be used with high success and low complication rates^[6,7]^.

Patients with pre-existing heart failure can be prone to acute hemodynamic compromise in the setting of sepsis due to endocarditis. In rare cases, short-term mechanical circulatory support can be necessary due to postcardiotomy syndrome. Since, in case of infection, all lead material must be removed, lead extraction during mechanical circulatory support can be necessary. If lead extraction by manual traction is not possible, treatment with powered extraction tools like laser or rotational mechanical sheaths may be necessary.

## TECHNIQUE

**Fig. 1:**
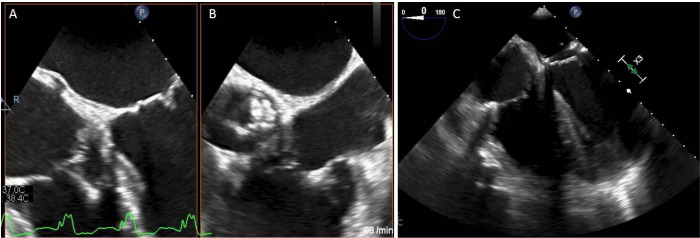
(A) Preoperative echocardiography showing RV lead with vegetation. (B) Echocardiographic image of the aortic valve with large endocarditis vegetation. (C) Intraprocedural transesophageal echocardiography (TEE) during lead extraction, showing complete removal of the RV lead and exclusion of pericardial effusion after lead extraction.

Lead extraction during venoarterial extracorporeal membrane oxygenation (VA-ECMO) support is a rare condition that requires some special techniques. We describe the technique in a 64-year old male patient with a history of aortic valve replacement and pacemaker dependency due to third-degree atrioventricular (AV) block, presenting with triple valve endocarditis including aortic, mitral and tricuspid valves. Furthermore, lead vegetation was present on the RV lead and the patient had a severely reduced left ventricular ejection fraction (LVEF) (Figure 1). He had one atrial lead and one active fixation ventricular lead, inserted through the left subclavian vein, implanted for 13 years. Due to the large vegetations in the aortic valve, the patient was treated emergently. The aortic valve was replaced by an Edwards Perimount 25 mm bioprosthesis, and the mitral and tricuspid valves were repaired with an IMR ETlogix 32 mm and a Medtronic Contour 3D 32 mm ring, respectively. The thrombi were removed from the RV lead and the leads were cut at the superior vena cava (SVC) level. Due to the pacemaker dependency, a right atrial and a right permanent ventricular epicardial lead were implanted. Due to the severely reduced LVEF and triple valve surgery, a weaning from cardiopulmonary bypass was not possible and the patient was placed on VA-ECMO via right subclavian artery and left femoral vein due to postcardiotomy syndrome. An attempt to extract the remaining leads from the subclavian route by manual traction with a stylet was not possible. Therefore, the patient was scheduled for a laser lead extraction procedure under VA-ECMO support the next day. The patient was treated under general anesthesia in a hybrid operating room (OR) using fluoroscopic guidance. The leads were prepared as previously described. The VA-ECMO flow was reduced to 1.5 l/min during lead extraction procedure to avoid collapse of the veins and prevent vascular damage.

**Fig. 2:**
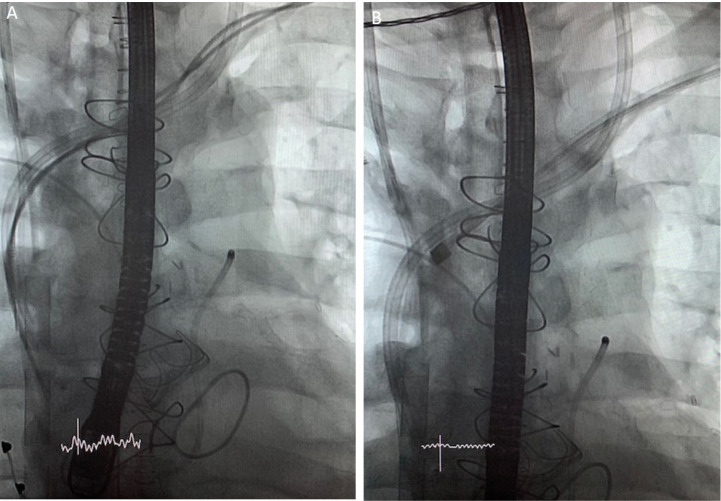
(A) Intraprocedural fluoroscopy showing 2 pacemaker leads that have been distally cut during an open surgical procedure and venous ECMO cannula ending in the right atrium. (B) Laser lead extraction procedure with RV lead in laser sheath.

**Fig. 3:**
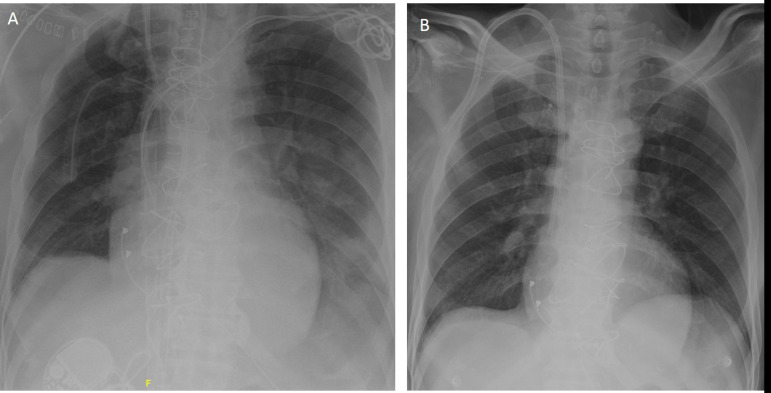
(A) Chest radiography after triple valve surgery, with remaining lead fragments and venous ECMO cannula in the right atrium. (B) Chest radiography after lead extraction and ECMO explantation.

After insertion of 2 lead locking devices (LLD), the laser lead extraction procedure was performed with 14-Fr GlideLight 80 Hz laser sheaths (Spectranetics Corporation, Colorado Springs, CO, USA) (Figure 2). Both leads were completely extracted, without complications, and extracorporeal membrane oxygenation (ECMO) flow was again increased to 5.0 liters (Figure 3). The patient was transferred back to the intensive care unit (ICU) and subsequently weaned from ECMO. After 6 days, ECMO could be successfully explanted. During hospital stay, the patient’s left ventricular function improved to 38%, so no ICD or cardiac resynchronization therapy (CRT) upgrade was necessary. After a long ICU stay, complicated by an abdominal ischemia, treated surgically, the patient recovered and could be discharged from the hospital.

## DISCUSSION

We have demonstrated a successful transvenous lead extraction during ongoing VA-ECMO support. In combination with surgical treatment of endocarditis and antibiotic therapy, a successful cure for systemic infection and sepsis was achieved.

Lead extraction in patients with systemic infection or lead-associated endocarditis is recommended in the lead management guidelines (class I)^[2-4]^. Complete removal of all lead material from vascular and antibiotic treatment is recommended^[8]^. In cases with valvular involvement, additional surgical valve repair or replacement is often necessary. Especially in cases of prosthetic valve endocarditis or cases with left-sided valvular vegetation, valve surgery is necessary to improve the prognosis of the patient^[5]^.

In cases with long lead implant duration, extraction by manual traction is often unsuccessful, and specialized lead extraction tools are necessary. Extraction by manual traction has been shown to have failure rates of up to 73%^[9]^. Even if the leads are cut at the SVC level and extraction is performed during open-heart surgery, manual traction alone does not often allow complete lead extraction, as previously described for patients after heart transplantation^[10]^. Since, in cases of systemic infection, the remaining lead fragments increase the risk of ongoing infection, complete lead extraction with specialized tools is of extraordinary importance in these cases. Furthermore, the enhanced visualization capacities of a hybrid OR with the possibility of multiplanar imaging are advantageous.

In the case of transvenous lead extraction in patients with short-term mechanical circulatory support devices like ECMO, some special considerations need to be taken into account. First, the venous ECMO cannula can hinder the progress of the laser/mechanical extraction sheath in the SVC during the extraction procedure. This can be especially an issue in patients with multiple leads and limited space in the SVC area. In some cases, repositioning of the venous ECMO cannula might be necessary. In cases with previous open-heart surgery, as in our patient, cutting the distal lead part as high as possible in the SVC can prevent from the above-mentioned issue.

Second, in patients with VA-ECMO support and high ECMO flow rates, there is a low filling of the venous system, which can lead to the collapse of the veins. This might increase the risk of venous laceration during the laser/mechanical extraction. Therefore, we recommend reducing the ECMO flow immediately before starting the extraction process, to allow the filling of the venous system and reduce the risk for damaging the veins with extraction tools. Depending on the hemodynamic ability of the patient, the ECMO flow can be reduced to 1-2 l/min for a short period of time. After successful extraction, the normal ECMO flow can be restored. The ECMO weaning should be initiated subsequently, when the hemodynamic situation of the patient is stabilized. In our case, a successful ECMO weaning was possible 6 days after lead extraction and additional antibiotic treatment for control of sepsis.

## CONCLUSION

We demonstrated our technique for lead extraction under VA-ECMO therapy. In the illustrated case, triple valve surgery, as well as removal of all infected lead material, led to a successful infection control and the patient was successfully weaned from ECMO.

**Table:** Authors' roles & responsibilities

v	Drafting the work or revising it critically for important intellectual content; final approval of the version to be published
JP	Substantial contributions to the conception or design of the work; or the acquisition, analysis or interpretation of data for the work; drafting the work or revising it critically for important intellectual content; final approval of the version to be published
TT	Substantial contributions to the conception or design of the work; or the acquisition, analysis or interpretation of data for the work; drafting the work or revising it critically for important intellectual content; final approval of the version to be published
CD	Substantial contributions to the conception or design of the work; or the acquisition, analysis or interpretation of data for the work; drafting the work or revising it critically for important intellectual content; final approval of the version to be published
HR	Drafting the work or revising it critically for important intellectual content; final approval of the version to be published
SP	Substantial contributions to the conception or design of the work; or the acquisition, analysis or interpretation of data for the work; drafting the work or revising it critically for important intellectual content; final approval of the version to be published
